# An Integrated Support and Information Centre in a Large U.K. Cancer Centre Established in 1993 and Replicated in More Than 60 Units Across the United Kingdom and Australia

**DOI:** 10.3747/co.v15i0.275

**Published:** 2008-08

**Authors:** E.J. Maher

**Affiliations:** Mount Vernon Cancer Centre, Northwood, Middlesex, U.K

**Keywords:** Cancer support, Macmillan, cam, complementary, patient-centered, survivorship

## Abstract

Established in 1993 after a 2-year consultation between professionals and cancer patients, the Lynda Jackson Macmillan Centre (ljmc) has been a catalyst for change in the United Kingdom. The Centre began with a small core staff in a purpose-built building next to a cancer centre, networking with outreach workers in 12 surrounding hospitals, with a mission to improve information, communication, and support for cancer patients. Since 1996, the ljmc model has been adopted and developed by the charity Macmillan Cancer Support and has been spread to more than 60 units across the United Kingdom and Australia.

Introducing complementary therapies (cams) to a cancer centre was a particular early challenge. Establishing a shared understanding of the role of complementary therapies and developing nationally accredited written information about them, credible recruitment and governance procedures for therapy practitioners, agreed outcome measures, and peer-reviewed evaluation and research have all been important in engaging cancer physicians and managers; however, charitable funding is still required to support free access to most complementary therapies.

An integrated supportive care service for cancer patients begins with a shift in the culture of cancer treatment organizations, moving from a professional-centred to a patient-centred agenda. Real reach and impact requires “new” ideas and services to be integrated into the routine practice of the cancer care delivery organizations. A key lesson learned over the last 15 years is that an integrated support centre must continually adapt to be viable. Sustaining meaningful user guidance is a particular challenge. Support for self-management and the testing and development of cam services are growing parts of the portfolio.

